# Country Indicators Moderating the Relationship Between Phubbing and Psychological Distress: A Study in 20 Countries

**DOI:** 10.3389/fpsyg.2021.588174

**Published:** 2021-12-24

**Authors:** Agata Błachnio, Aneta Przepiórka, Oleg Gorbaniuk, Monika McNeill, Rebecca Bendayan, Mithat Durak, Emre Senol-Durak, Menachem Ben-Ezra, Martina Benvenuti, Alan Angeluci, Ana Maria Abreu, Meiko Makita, María J. Blanca, Tihana Brkljacic, Nenad Č. Babič, Julia Gorbaniuk, Juraj Holdoš, Ana Ivanova, Sadia Malik, Anita Milanovic, Bojan Musil, Igor Pantic, Belén Rando, Gwendolyn Seidman, Lancy D’Souza, Mariek M. P. Vanden Abeele, Mariusz Wołońciej, Anise M. S. Wu, Shu Yu, Elvis Mazzoni

**Affiliations:** ^1^Institute of Psychology, The John Paul II Catholic University of Lublin, Lublin, Poland; ^2^Glasgow Caledonian University, Glasgow, United Kingdom; ^3^Department of Biostatistics and Health Informatics, Institute of Psychiatry, Psychology and Neuroscience, King’s College London, London, United Kingdom; ^4^Department of Psychobiology and Methodology for Behavioral Sciences, University of Málaga, Málaga, Spain; ^5^Bolu Abant Izzet Baysal University, Bolu, Turkey; ^6^Ariel University, Ariel, Israel; ^7^Department of Psychology, University of Bologna, Bologna, Italy; ^8^Universidade Municipal de São Caetano do Sul, São Caetano do Sul, Brazil; ^9^Centre for Interdisciplinary Research in Health, Institute of Health Sciences, Universidade Católica Portuguesa, Lisbon, Portugal; ^10^University of Wolverhampton, Wolverhampton, United Kingdom; ^11^Institute of Social Sciences Ivo Pilar, Zagreb, Croatia; ^12^Department of Psychology, University of Maribor, Maribor, Slovenia; ^13^Catholic University in Ružomberok, Ružomberok, Slovakia; ^14^Department of Psychology, University of Sargodha, Sargodha, Pakistan; ^15^Clinic for Mental Disorders “Dr Laza Lazarević”, Belgrade, Serbia; ^16^Faculty of Medicine, University of Belgrade, Belgrade, Serbia; ^17^Institute of Social and Political Sciences, University of Lisbon, Lisbon, Portugal; ^18^Albright College, Reading, PA, United States; ^19^Faculty of Medicine, University of Mysore, Mysore, India; ^20^Department of Cognition and Communication, Tilburg University, Tilburg, Netherlands; ^21^Imec-mict-UGent, Ghent University, Ghent, Belgium; ^22^Department of Psychology, University of Macau, Taipa, China

**Keywords:** country indicators, culture, phubbing, mobile phone addiction, distress

## Abstract

Problematic mobile phone use can be related to negative mental states. Some studies indicate that behavioural dependency is related to variables associated with the country of origin. The aim of our study was to investigate if country indicators moderated the relationship between phubbing and psychological distress. Our sample consisted of 7,315 individuals from 20 countries, who completed the Phubbing Scale and the Kessler Psychological Distress Scale (K6). The analyses also included country indicators: the Gender Gap Index (GGI), the Human Development Index (HDI), the Social Progress Index (SPI), Hofstede’s dimensions of culture, and the World Happiness Index (WHI). Our results showed that psychological distress was related to at least one dimension of phubbing (i.e., to communication disturbance or phone obsession) in all countries, which means this relationship is culturally universal. The results of the study demonstrate the importance of testing measurement invariance to determine what type of analysis and what type of conclusion are valid in a given study or comparison. Moreover, the increasing or decreasing correlation between phubbing and distress is related to some culture-level indices.

## Introduction

A great body of research reports the increasing use of mobile phones (e.g., Lopez-Fernandez et al., 2017; Al-Saggaf and MacCulloch, 2019). Mobile phones are preferred for social media use and communication purposes, particularly, when face-to-face contact is not practical or possible (Karadağ et al., 2015; Chotpitayasunondh and Douglas, 2016), but their use is leading to interpersonal problems. A new phenomenon has emerged recently, whereby phone users ignore other people around them by using their mobile phones instead (Karadağ et al., 2015; Abeele, 2019; Abeele et al., 2019; Balta et al., 2020). This phenomenon is called “phubbing,” and its name is derived from two words: “phone” and “snubbing.” Phubbing behaviour is typically seen among individuals who are distracted by their phone when it is not ringing or vibrating and who are not paying attention to others around them (Afdal et al., 2019). It also manifests itself in people preferring to pay attention to smartphones rather than to their interlocutor during face-to-face interaction (Chotpitayasunondh and Douglas, 2016), such as interaction with their close family members (Al-Saggaf and MacCulloch, 2019). Phubbing can be called *absent presence* when a person uses their mobile phone in the company of others (Abeele et al., 2016). It may disrupt interpersonal relationships (Ergün et al., 2020), and people engaging in it may show withdrawal symptoms when they are away from their phones (Karadağ et al., 2015). Research has shown that other types of problematic technology use, such as Internet addiction, mobile phone addiction, mobile game addiction (Chotpitayasunondh and Douglas, 2016, 2018; T’ng et al., 2018), and fear of missing out, can lead to phubbing. Phubbing in turn often leads to violations of cultural values and to disrespectful attitudes, regardless of culture (Afdal et al., 2019).

### Phubbing and Psychological Distress

Apart from the changes mentioned above, researchers have investigated the possible causes of phubbing behaviour. It has been highlighted that phubbing is a way of coping with loneliness (Jackson and Wang, 2013; Karadağ et al., 2015; Afdal et al., 2019), worry, and anxiety and that it is related to deprivation in situations when one is far from one’s phone (Karadağ et al., 2015). Phubbing is positively correlated with anxiety (Khare and Qasim, 2019) and has an impact on interpersonal relationships and personal wellbeing (WB) (Roberts and David, 2016). A high level of partner phubbing is related to depression and low relationship satisfaction (Wang et al., 2017); it also has a negative impact on intimacy (Abeele et al., 2019).

Another possible cause of phubbing behaviour is psychological distress, which is an indicator of mental health (Kessler et al., 2003). Psychological distress is defined as a state of emotional suffering characterized by inefficiency in coping, feelings of discomfort, and changes in emotional WB (Walker and Avant, 1995), such as moderate-to-severe symptoms of anxiety and depression (Drapeau et al., 2012). Phubbing has also been found to function as a mediator between phone addiction and depression (Ivanova et al., 2020). In a different study, higher partner phubbing was correlated with lower life satisfaction and higher depression scores (Roberts and David, 2016). The experience of psychological distress was found to be related to uncertain social relations and time pressure (Türetgen et al., 2012), anxiety (Tan and Lau, 2012), and phubbing (Lian et al., 2021).

Some studies have revealed that individuals with better mental health are more likely to exhibit lower levels of phubbing behaviour (Babadi-Akashe et al., 2014). Another study showed that rumination mediated the relationship between psychological distress and phubbing among adolescents (Lian et al., 2021). Despite a Pakistani study indicating that phubbing is directly related to distress (Shahbaz et al., 2020), other researchers suggest moderator and mediator variables between psychological distress and phubbing (Lian et al., 2021). Being phubbed increases the levels of depression and stress (David and Roberts, 2017) and is related to lower self-flourishing (Davey et al., 2018). Adolescents who are phubbed by their mothers feel ignored, and the quality of their relationship with their mother decreases (Bai et al., 2020). This can be explained by the diathesis-stress model (Monroe and Simons, 1991), which underlines the fact that psychological disorders may be a result of the interaction between genetic predisposition, vulnerability, and stress triggered by life events. This model points toward certain moderators, such as personal traits, in the relationship between the environmental factors and the development of a psychological disorder (Monroe and Simons, 1991). Therefore, exploring conditional variables associated with psychological distress and phubbing is considered to be important for the understanding of the circumstances correlated with those variables. In the present study, we focused on the moderating role of country, since cultural differences have proved to be important in phubbing (Al-Saggaf and MacCulloch, 2019).

### Country Indicators

Social norms play a considerable role in phubbing (Al-Saggaf and MacCulloch, 2019). Some studies have shown that people from different cultures use new media in different ways. For instance, it was found that people from individualistic countries preferred using social networking sites (SNSs) actively (e.g., Jackson and Wang, 2013; Makri and Schlegelmilch, 2017), while people from collectivistic cultures used SNSs to receive social support and strengthen social connections (Jackson and Wang, 2013; LaRose et al., 2013), people from individualistic cultures use SNSs to satisfy their individual needs, such as the need to escape from loneliness (Jackson and Wang, 2013). A meta-analysis, whose authors checked moderated effect of culture in the relation between SNSs and mental health, revealed that the relations between SNS use and mental health in collectivistic cultures were stronger than in individualistic cultures (Yin et al., 2019). Additionally, Arpaci (2019) reported that there was a link between vertical collectivism and nomophobia—a fear of not having access to one’s mobile phone.

Some studies indicate that behavioural dependency is related to country variables. For instance, some researchers suggest that cultural dimensions determine SNS usage patterns. Vasalou et al. (2010) found differences in Facebook use across different countries. For example, users from the United Kingdom spent more time on Facebook than users from other countries. Participation in Facebook groups was more important for United Kingdom users than for United States users, while Italians preferred participating in groups and playing games. In Greece, users regarded updating their profiles as the least important activity in comparison with other countries (Vasalou et al., 2010). Culture can be a predictor of online and technology-related behaviours (Chau et al., 2002; Arpaci, 2019; Błachnio et al., 2019a). For instance, previous studies indicated that people from vertical (as opposed to horizontal) collectivistic cultures, which promote sacrificing oneself in relationships, had a greater tendency to develop nomophobia (Arpaci, 2019). Yin et al. (2019) established the moderating role of cultural background in the relationship between SNS use and positive mental health. A meta-analysis carried out by Zhang et al. (2012) showed that culture had a moderating effect on mobile commerce adoption. Another study showed that collectivism, uncertainty avoidance (UAI), short-term orientation, and power distance could be cultural moderators for the use of mobile technologies (Baptista and Oliveira, 2015). Research results highlighted the moderating effect of cultural dimensions, such as UAI, individualism, and long-term orientation, on the adoption of novel mobile services (Hung and Chou, 2014).

Understanding how phubbing behaviour is influenced by social circumstances seems to be important for professionals dealing with social life problems (Chotpitayasunondh and Douglas, 2016). Social motives and accessibility to mobile technologies are of significance in phubbing behaviour (e.g., Jackson and Wang, 2013). In an attempt to determine intercultural differences in phubbing as has been done for Facebook use (e.g., by Ji et al., 2010) and for SNS use (e.g., by Jackson and Wang, 2013), we drew on Hofstede’s (1980) understanding of culture. Hofstede describes the culture in terms of dimensions, such as individualism vs. collectivism, UAI, power distance, or masculinity vs. femininity^1^.

We predicted that in different countries, phubbing would correlate with distress to different degrees. Based on previous studies indicating that phubbing is negatively related to mental health (Karadağ et al., 2015; Khare and Qasim, 2019; Ivanova et al., 2020), we hypothesised that phubbing would be positively related to distress. Different cultures differ in the extent to which mental health is dependent on the quality of social relationships, the role of a social group, and social support from the group one belongs to De Silva et al. (2007). Because phubbing involves the disruption of communication with others as a result of using a mobile phone in their presence, it leads to the deterioration of social relationships, which in turn may have different consequences for mental health in different cultures. We predicted that in those cultures where the importance attached to social relationships was greater phubbing would have a stronger impact on mental WB (distress). We therefore examined the role of culture in this relationship to investigate whether country indicators were moderators between phubbing dimensions and psychological distress. Specifically, we selected the following indicators: the Gender Gap Index (GGI), the Social Progress Index (SPI), the World Happiness Index (WHI), the Human Development Index (HDI), and Hofstede’s cultural value indices.

While mobile phones enable communication and facilitate many activities in everyday life (Luo and Tuney, 2015; Chotpitayasunondh and Douglas, 2016; Karadağ et al., 2015), their excessive use has negative consequences as well (Lopez-Fernandez et al., 2017). The main aim of our research was to explore the moderating role of the cultural specificities of countries in the relationship between phubbing and psychological distress. The study fills a gap in the knowledge regarding the role that cross-national differences play in this relationship. On the one hand, the inclusion of cross-national moderators is exploratory and aimed at identifying the cultural characteristics that play an important role in the relationship between phubbing and distress. On the other hand, testing these characteristics as potential moderators is warranted by the results of previous research.

Cultural indicators differentiate countries on different dimensions (see Bleidorn et al., 2015; Jonason et al., 2020). Previous studies have shown that different dimensions of culture can play moderating roles (e.g., Sutrisno and Dularif, 2020). The socioeconomic context was found to be a moderator in the relationship between depression and body mass index (Alvarez-Galvez and Gomez-Baya, 2017). Moreover, country indices have proved to be significant moderating factors for the level of Internet addiction (Błachnio et al., 2019b).

Countries differ in the availability (equal or unequal) of various resources and opportunities to women and men and in the status of the two genders (GGI; Bosson et al., 2021). This means that in countries with low GGI, men are more dominant while women are more subordinate (Bosson et al., 2021). In countries where the role of women is subordinate (low GGI), a weaker association between phubbing and distress can be expected. Other indices differentiate countries in terms of general WB and quality of life. The SPI shows how countries differ in terms of the real quality of life, which is closely related to the economic level and thus to the satisfaction of needs or the fulfilment of opportunities. Countries also differ in the levels of human potential, WB, life expectancy, economic growth, and access to education, reflected in the HDI. The higher the HDI, the more a country is perceived to meet the basic needs of its citizens and the more autonomy it offers in various social choices, such as work or education (Bosson et al., 2021). Previous studies have shown strong relationships between GGI and HDI (Bosson et al., 2021), which make it reasonable to predict HDI levels similar to those of GGI. The next index, the WHI differentiates countries in terms of global happiness. Research shows that countries with high WHI are those where support and WB, such as income, healthy life expectancy, social support, freedom, trust, and generosity, are key factors (see World Happiness Report), which may translate into a higher correlation between phubbing and distress in these countries.

On the one hand, it can be expected that countries with greater access to mobile phones will be more likely to have higher rates of problematic phone use and greater distress levels associated with it. On the other hand, in less developed countries, phubbing may lead to greater distress, as the people there are less accustomed to the presence of phones in social life. The indicators that differentiate countries also include the Hofstede dimensions (we chose only those indices that had a full set of values for all the countries investigated): individualism vs. collectivism, masculinity vs. femininity, and UAI. Previous studies have used Hofstede dimensions to explain differences in the Internet or Facebook use (e.g., Nadkarni and Hofmann, 2012; Jackson and Wang, 2013; Abbas and Mesch, 2015) but these dimensions were not tested as moderators of the relationship between new technology addiction and mental health. In our study, we posed the question of how these dimensions might differentiate the relationship between phubbing and distress across countries. It could only be speculated, for example, that phubbing would lead to distress to a lesser extent in individualistic countries.

## Materials and Methods

### Participants and Procedure

A sample of *N* = 7,315 mobile phone users (66.4% women and 33.6% men) was recruited for the study. Data were collected online in 20 countries: Brazil, China, Croatia, Ecuador, India, Israel, Italy, Mexico, Netherlands, Pakistan, Poland, Portugal, Serbia, Slovakia, Slovenia, Spain, Turkey, the United Kingdom, Ukraine, and the United States. The mean age of the total sample was *M* = 25.50 years (*SD* = 9.66; range: 16–85 years). Out of the total number of the participants, 79.3% were students (16.0% of them working students), 17.9% were employed, 1.7% were unemployed, and 1.1% were retired.

The individuals invited to participate in the study were mobile phone users. The study was conducted in local languages, and back-translation procedures were applied to adapt the measures. We used snowball sampling as a method of reaching a large group of respondents, varied in terms of sociodemographic characteristics. After the electronic versions of the questionnaires were prepared, the link to the research site was e-mailed to participants. The participants volunteered to take part in the study and received no monetary reward. They were informed about the anonymity of the study. The research project was approved by the institutional review board at the university of the first author.

It is important to note that 3.45% of the participants were failed to provide more than 10% of the answers and were therefore excluded from the sample. However, 0.88% of the participants were failed to provide less than 10% of answers; they were included in the sample and in the analysis. Their scores were extrapolated from their other responses on a given scale; any missing data were randomised. We found no significant effects for age and gender.

### Measures

#### Psychological Distress

Kessler Psychological Distress Scale (K6) was used to measure psychological distress (Kessler et al., 2003). The scale consists of six questions concerning depressive and anxiety-related symptoms that a person have experienced in the past 4 weeks (e.g., “Did you feel tired for no good reason?”). Cronbach’s alpha for the K6 ranged from 0.74 (India) to 0.90 (United Kingdom).

Prior to hypothesis testing, we assessed the measurement invariance of the K6 across countries using multi-group confirmatory factor analysis (MGCFA). We found only metric invariance for the scale (Table 1) according to the criteria proposed by Rutkowski and Svetina (2014). This means that we could not compare means on Level 2 and establish its country-level correlates, but we could compare correlations between variables across countries (Milfont and Fischer, 2010) and identify country-level moderators. Additionally, testing measurement invariance across age groups and genders revealed metric invariance across age groups and scalar invariance across genders (see Table 1).

**TABLE 1:** Testing of measurement invariance for Distress Scale across countries, age categories and gender.

	Country			Age			Gender		
Invariance	χ ^2^ (*df*)	CFI	RMSEA	χ ^2^ (*df*)	CFI	RMSEA	χ ^2^ (*df*)	CFI	RMSEA
Configural	750.55(160)*	0.966	0.023	381.75(16)*	0.975	0.058	434.27(16)*	0.974	0.050
Metric	1237.09(255)*	0.946	0.023	419.08(21)*	0.973	0.053	441.96(21)*	0.974	0.053
Scalar	4203.94(369)*	0.789	0.038	784.05(27)*	0.949	0.064	617.32(27)*	0.964	0.055
Configural vs. metric	486.54(95)*	0.020	0.000	37.33(5)*	0.002	0.005	7.69(5)	0.000	0.003
Metric vs. scalar	2966.85(114)*	0.157	0.015	364.97(6)*	0.024	0.011	175.36(6)*	0.010	0.002

*χ^2^ tests are significant at p < 0.001.

#### Phubbing

The Phubbing Scale (Karadağ et al., 2015) was also used in the study. The items of the scale were chosen based on other technology-related addictions (i.e., Internet addiction, SMS addiction, and social media addiction). Exploratory and confirmatory factor analyses revealed a two-factor structure of the measure. The questionnaire consists of eight items (e.g., “My eyes start wandering on my phone when I’m together with others”; “People complain about me dealing with my mobile phone”) rated on a 5-point scale (1 = *strongly disagree* to 5 = *strongly agree*). The measure comprises two factors: (1) communication disturbance, defined as disturbing face-to-face communication by dealing with one’s mobile phone (four items), and (2) phone obsession, defined as constantly needing and desiring one’s mobile phone (4 items).

Based on data collected in 20 countries, Cronbach’s alpha ranged from 0.71 (India) to 0.95 (Serbia) for the Communication Disturbance Scale and from 0.66 (Slovenia) to 0.81 (Serbia) for the Phone Obsession Scale. The results of measurement invariance for the Phubbing Scale across 20 countries, age groups, and genders are presented in Table 2. There was only metric invariance across countries and age groups and scalar invariance across genders. This allowed us to compare countries in terms of correlations between phubbing and other variables and identify country-level moderators of these relationships.

**TABLE 2:** Testing of measurement invariance for Phubbing Scale across countries, age categories and gender.

	Country			Age			Gender		
Invariance	χ ^2^ (*df*)	CFI	RMSEA	χ ^2^ (*df*)	CFI	RMSEA	χ ^2^ (*df*)	CFI	RMSEA
Configural	1274.45(380)*	0.952	0.018	1059.25(38)*	0.941	0.063	1098.49(38)*	0.942	0.062
Metric	1679.89(494)*	0.936	0.018	1076.16(44)*	0.940	0.059	1103.98(44)*	0.942	0.058
Scalar	7150.84(646)*	0.648	0.037	1409.44(52)*	0.921	0.062	1242.87(52)*	0.934	0.056
Configural vs. metric	405.45(114)*	0.016	0.000	16.91(6)	0.001	0.004	5.49(6)	0.000	0.004
Metric vs. scalar	5470.94(152)*	0.288	0.019	333.28(8)*	0.019	0.003	138.89(8)*	0.008	0.002

*χ^2^ tests are significant at p < 0.001.

#### Cultural Indicators

We included cultural-level indicators in the analysis, choosing the indicators previously used in other studies (Bleidorn et al., 2015; Błachnio et al., 2019b; Jonason et al., 2020). We relied on the latest data comparisons available and used the following indices (the specific scores for all countries are presented in Table 3):

***The GGI*** is an indicator of national gender gaps in economic participation, educational attainment, political empowerment, and health and survival criteria. GGI data are published annually, and we retrieved the 2020 GGI scores (Global Gender Gap Report, 2020). An index for each country is between 0 and 1. In the present sample, GGI values ranged from 0.56 (Pakistan) to 0.80 (Spain); *M* = 0.71, *SD* = 0.05.

***The SPI*** is a measure of the real quality of life, which is independent of economic indicators. It comprises three aspects: basic human needs (HN), foundations of WB, and opportunity (O). SPI values ranged from 48.2 (Pakistan) to 88.3 (Netherlands); *M* = 76.42, *SD* = 10.66. The values for specific aspects were as follows: from 58.5 (Pakistan) to 96.7 (Netherlands) for basic HN (*M* = 87.1, *SD* = 9.7); from 48.8 (Pakistan) to 90.3 (Turkey) for foundations of WB (*M* = 77.5, *SD* = 10.8); and from 37.3 (Pakistan) to 80.3 (United Kingdom) for O (*M* = 0.82, *SD* = 0.09).

***The WHI*** measures the state of global happiness and ranks countries according to their happiness level. We retrieved data from the 2019 comparison.^2^ The WHI values for the countries included in the study were ranged from 4.02 (India) to 80.3 (Netherlands); *M* = 5.99, *SD* = 0.85.

***The HDI*** covers three dimensions of human development, namely, living a long and healthy life, being educated, and having a decent standard of living. The values of HDI range between 0 and 1, with higher values indicating higher human development. We retrieved data from the 2019 comparison^3^. In the present sample, HDI values ranged from 0.56 (Pakistan) to 0.93 (Netherlands); *M* = 0.82, *SD* = 0.09.

***Hofstede’s cultural value indices*** were also used in our study. We selected three cultural value dimensions identified by Hofstede^4^. The scores on each dimension range between 0 and 100. *Individualism* vs. *collectivism (IND)*: individualism is a feature of those cultures where people are expected to care only for themselves and their immediate families, while in collectivistic cultures people take care of their relatives and are loyal to their community. In the present sample, IND scores ranged between 8 (Ecuador) and 91 (United States), with higher scores indicating greater individualism (*M* = 44.25, *SD* = 23.99). *Masculinity vs. femininity (MAS):* Masculinity manifests itself in the following characteristics of a society: achievement, heroism, assertiveness, and material rewards for success. Femininity is marked by a preference for cooperation, modesty, caring for the weak, and preoccupation with quality of life. In the present sample, MAS scores ranged between 14 (Netherlands) and 100 (Slovakia), with higher scores indicating greater masculinity (*M* = 51.15, *SD* = 19.60). *Uncertainty avoidance (UAI)* was the final cultural value indicator used in our study. It indicates the degree to which people in a society feel uncomfortable with uncertainty and ambiguity regarding the future. Societies scoring higher on UAI are more emotional and less open to change. In the present sample, UAI scores ranged between 30 (China) and 99 (Portugal); *M* = 71.20, *SD* = 20.79.

**TABLE 3:** Sample characteristics, country indicators, and correlation of distress with phubbing and phone obsession within each country.

				Within-country variables															
		Male	Age	Phubbing	Obsession	Distress	Cultural indicators										Within-country correlations		
Country	*N*	%	*M* (*SD*)	*M* (*SD*)	*M* (*SD*)	*M* (*SD*)	GGI	HDI	SPI	HN	WB	O	IND	MAS	UAI	WHI	Ph-Dist	Obs-Dist	Ph-Obs
Brazil	311	46.6	23.52(6.05)	2.03(0.76)	3.61(0.92)	2.48(0.80)	0.691	0.761	72.87	81.79	76.56	60.26	38	49	76	6.300	0.22**	0.24**	0.44**
China	401	20.2	−	2.19(0.63)	3.66(0.86)	2.27(0.81)	0.676	0.758	64.54	81.35	68.85	43.41	20	66	30	5.191	0.08*	0.07	0.17**
Croatia	688	47.4	21.81(2.38)	1.92(0.68)	3.30(0.83)	2.24(0.75)	0.720	0.837	79.21	90.90	80.88	65.86	33	40	80	5.432	0.22**	0.18**	0.44**
Spain	511	42.9	30.16(12.66)	2.17(0.72)	2.96(0.81)	2.17(0.77)	0.795	0.893	87.47	94.77	69.97	77.30	51	42	86	6.354	0.15**	0.22**	0.50**
Netherlands	271	42.5	44.25(18.00)	2.18(0.67)	3.23(0.76)	1.68(0.64)	0.736	0.933	88.31	96.74	88.30	76.12	80	14	53	7.488	0.20**	0.14*	0.50**
Israel	390	38.2	37.32(12.33)	2.59(0.93)	3.29(0.96)	1.86(0.73)	0.718	0.906	81.44	93.58	84.46	66.27	54	47	81	7.139	0.17**	0.01	0.58**
Mexico	57	19.3	39.44(9.83)	2.89(0.86)	3.64(0.67)	1.85(0.67)	0.754	0.767	77.51	82.31	74.67	57.54	30	69	82	6.595	0.18	0.09	0.41**
Pakistan	410	30.0	22.31(3.72)	2.35(0.78)	3.21(0.90)	2.74(0.79)	0.564	0.560	48.20	58.46	48.83	37.29	14	50	70	5.653	0.24**	–0.02	0.37**
Poland	406	20.6	23.51(5.06)	1.62(0.59)	2.81(0.90)	2.31(0.79)	0.736	0.872	81.25	94.11	81.00	68.65	60	64	93	6.182	0.13**	0.22**	0.44**
Portugal	400	33.8	26.08(8.76)	2.21(0.68)	3.04(0.89)	2.25(0.78)	0.744	0.850	87.12	95.81	87.43	78.12	27	31	99	5.693	0.16**	0.18**	0.52**
Serbia	365	37.0	26.17(5.60)	2.26(1.13)	3.28(0.89)	2.26(0.65)	0.736	0.799	71.59	86.00	70.97	75.58	25	43	92	5.603	0.19**	0.23**	0.42**
Slovenia	430	21.4	22.13(4.53)	1.97(0.67)	3.11(0.76)	2.14(0.71)	0.743	0.902	85.80	95.64	86.18	75.81	27	19	88	6.118	0.20**	0.26**	0.47**
United States	190	18.2	20.98(5.26)	2.37(0.71)	3.35(0.79)	2.32(0.80)	0.724	0.920	83.62	91.64	82.05	77.17	91	62	46	6.892	0.12*	0.23**	0.39**
Italy	603	17.7	22.28(4.30)	1.96(0.58)	3.27(0.81)	2.39(0.81)	0.707	0.883	85.69	92.32	88.64	79.88	76	70	75	6.223	0.17**	0.20**	0.41**
Ukraine	402	24.9	20.96(3.36)	1.76(0.58)	2.91(0.95)	2.38(0.82)	0.721	0.750	66.97	82.21	64.22	54.47	25	27	95	4.332	0.10*	0.12**	0.45**
India	126	47.6	25.28(8.03)	2.15(0.82)	2.60(1.00)	2.31(0.76)	0.668	0.647	59.10	67.72	58.94	50.63	48	56	40	4.015	0.32**	0.36**	0.46**
United Kingdom	135	15.6	32.03(14.07)	1.98(0.68)	3.26(0.92)	2.54(0.97)	0.767	0.920	87.98	94.63	89.05	80.28	89	66	35	7.054	0.34**	0.25**	0.46**
Slovakia	181	60.0	24.95(8.98)	1.89(0.65)	3.09(0.86)	2.36(0.83)	0.718	0.857	80.43	94.04	80.97	66.29	52	100	51	6.198	0.17*	0.12	0.37**
Ecuador	415	33.5	21.87(4.26)	1.83(0.67)	2.61(0.90)	2.37(0.71)	0.729	0.758	71.88	82.57	77.01	56.05	8	63	67	6.028	0.23**	0.22**	0.50**
Turkey	623	28.1	23.55(6.52)	2.37(0.67)	3.44(0.84)	2.60(0.76)	0.635	0.806	67.49	85.00	90.34	47.50	37	45	85	5.373	0.28**	0.23**	0.46**

GGI = Global Gender Gap Index; HDI = Human Development Index; SPI = Social Progress Index; HN = Basic Human Needs; WB = well-being; O = Opportunity; IND = individualism vs. collectivism; MAS = Masculinity vs. Femininity; UAI = Uncertainty Avoidance; WHI = World Happiness Index; UK = United Kingdom; USA = United States of America; Dist = Distress; Ph = Phubbing; Obs = Obsession. P-value for two-tailed test * p < 0.05, ** p < 0.01.

### Statistical Analyses

For the primary analyses, we conceptualised data as a two-level structure, in which individual respondents were nested within countries of residence. We used Mplus 7.3 software (Muthén and Muthén, 2015) to analyse a series of multilevel models (MLM). These analyses were conceptually equivalent to conducting a regression analysis for each country and then using the coefficients thus estimated as dependent measures at the next level of analysis. Level 1 represented variation among individuals within countries, and Level 2 represented variation across the 20 countries.

The relationships between psychological distress, communication disturbance, and phone obsession were examined at the individual level, and the country-level differences in these relationships were modelled at the between-country level as a function of the cultural specificity of a given country. Analyses examining such relationships are called *slopes-as-outcomes* analyses because a slope from a lower level (i.e., Level 1) becomes an outcome at an upper level (i.e., Level 2).


(1) Psychological_distress=ijβ+0jβ(Communication_disturbance)ij1j+β(Phone_obsession)ij2j+r.ij

(2) β=0jγ+00γ(MODERATOR)j01+u.0j

(3) β=1jγ+10γ(MODERATOR)j11+u.1j

(4) β=2jγ+20γ(MODERATOR)j21+u.2j

In Eq. 1, Level-1 observations (psychological distress*_ij_*) are modelled as a function of the intercept for each country (β_0_*_j_*, mean psychological distress in a country *j*), the slopes (e.g., β_1_*_j_*, representing a within-country relationship between psychological distress and communication disturbance), and error (r*_ij_*, which is the deviation of each psychological distress score in a country from the country mean), and the variance in *r*_*ij*_ is Level-1 error variance.

In Eq. 2, mean psychological distress for each of *j* Level-2 units of analysis (i.e., countries; β_0_*_j_*) is modelled as a function of the grand mean (γ_00_ = the mean of psychological distress means), country specificity (γ_01_ MODERATOR*_j_*) and error (u_0_*_j_*), and the variance in u_0_*_j_* is the Level-2 variance. If the γ_01_ coefficient is significantly different from zero, then there is a relationship between a country index and the average psychological distress score for people in *j* country.

In Eq. 3 (or 4), the Level-1 slope for each country (β_1_*_j_* or β_2_*_j_*) is modelled as a function of the intercept (γ_10_ or γ_20_ = the mean slopes, i.e., the average relationship across all countries), country cultural specificity (γ_11_ MODERATOR*_j_* or γ_21_ MODERATOR*_j_*), and error (u_1_*_j_*). If the γ_11_ (or γ_21_) coefficient is significantly different from zero, then the relationship between psychological distress and communication disturbance (or between psychological distress and phone obsession) varies as a function of country cultural specificity (MODERATOR*_j_*).

Due to the lack of scalar invariance across 20 countries in measures of distress, communication disturbance, and phone obsession, β_0_*_j_* could not be modelled as a function of differences between countries β_0_*_j_* = γ_00_ + γ_01_ (MODERATOR*_j_*) + μ_0_*_j_*. However, we were able to model the correlations between variables in different countries and the cultural moderators of these relationships. This is the reason why Level-1 variables were group mean-centred; consequently, Level-2 differences in these Level-1 variables were eliminated from the model. In addition, to simplify the interpretation of the regression equation, we applied the standardized country indicators at the country level (“z”). After the centring of the variables within cluster (“c”) at Level 1, the mode equations are as follows:


(5) cPsychological_distress=ijβ(cCommunication_disturbance)ij1j+β(cPhone_obsession)ij2j+r.ij

(6) β=1jγ+10γ(zMODERATOR)j11+u.1j

(7) β=2jγ+20γ(zMODERATOR)j21+u.2j

## Results

### Descriptive Statistics

Table 3 provides the basic statistics for each country: means and SDs for psychological distress, communication disturbance, phone obsession, and cultural indicators; it also presents correlations between the dimensions of psychological distress and phubbing within each country. These summary statistics constitute a context for the analyses focused on the primary hypothesis.

The correlation between psychological distress and communication disturbance was positive and ranged between 0.08 (China) and 0.34 (United Kingdom), while the correlation between psychological distress and phone obsession ranged between −0.02 (Pakistan) and 0.36 (India).

### Slope-as-Outcome Models: Culture-Level Moderators

First, the predictors of psychological distress at Level 1 (i.e., communication disturbance and phone obsession)—centred group means—were entered with a random error term according to Eqs 6, 7 but without cross-cultural moderators (zMODERATOR*_j_* = 0). Significance tests at Level 2 (between countries) showed that γ_10_ and γ_20_ coefficients, representing the mean slopes between psychological distress and communication disturbance (γ_10_ = 0.153, 95% CI [0.113, 0.192]) and between psychological distress and phone obsession (γ_10_ = 0.098, 95% CI [0.052, 0.144]), were significantly different from 0 and positively related to psychological distress.

Next, we analysed the previous regression model at Level 1 by including the z-standardized cultural indicators as explanatory variables at Level 2. We were interested in explaining the observed cross-cultural variation in regression coefficients at Level 1. That is, we were not interested in explaining cross-cultural mean-level differences in psychological distress, communication disturbance, and phone obsession due to the lack of scalar measurement invariance of these variables across countries. Each cultural indicator was entered separately as an explanatory variable in the slope-as-outcome models. The coefficients are presented in Table 4 (left panel). These parameters may inform the question of what cultural indicators explain the cross-cultural variation in regression coefficients at Level 1.

**TABLE 4:** Slope-as-outcome models: Cross-level moderations and mean slopes of the communication disturbance and phone obsession effects on the distress.

	Cross-level moderation of the relationship between:				Mean slope of the relationship between:			
	Communication disturbance – distress		Phone obsession – distress		Communication disturbance – distress		Phone obsession – distress	
Moderator	γ _11_	95% CI	γ _21_	95% CI	γ _10_	95% CI	γ _20_	95% CI
(lack)	−	−	−		0.153	0.113;0.192	0.098	0.052;0.144
GGI	−0.044**	−0.076;−0.011	0.042*	0.000;0.083	0.147	0.113;0.182	0.103	0.059;0.145
HDI	–0.028	−0.066;0.013	0.037	−0.009;0.082	0.151	0.112;0.191	0.099	0.054;0.143
SPI	–0.028	−0.065;0.012	0.038*	−0.007;0.080	0.150	0.111;0.190	0.100	0.056;0.143
HN	−0.033*	−0.070;0.006	0.038*	−0.007;0.081	0.151	0.113;0.190	0.099	0.054;0.142
WB	–0.001	−0.041;0.041	0.029	−0.016;0.074	0.152	0.111;0.194	0.098	0.052;0.143
O	−0.035*	−0.070;0.004	0.046*	0.004;0.088	0.150	0.114;0.188	0.100	0.057;0.142
IND	–0.003	−0.046;0.043	0.032	−0.016;0.079	0.152	0.110;0.194	0.100	0.053;0.145
MAS	0.012	−0.034;0.058	–0.003	−0.055;0.047	0.154	0.112;0.196	0.098	0.048;0.145
UAI	–0.026	−0.070;0.017	0.000	−0.051;0.048	0.156	0.115;0.197	0.097	0.049;0.145
WHI	0.007	−0.037;0.053	–0.014	−0.066;0.035	0.153	0.112;0.195	0.097	0.048;0.145

GGI = Global Gender Gap Index; HDI = Human Development Index; SPI = Social Progress Index; HN = Basic Human Needs; WB = well-being; O = Opportunity; IND = individualism vs. collectivism; MAS = masculinity vs. femininity; UAI = uncertainty avoidance; WHI = World Happiness Index. p-value for one-tailed test: *p < 0.05, **p < 0.01.

As can be seen from the coefficients in Table 4, the GGI (γ_11_ = −0.044, *p* < 0.01), HN (γ_11_ = −0.033, *p* < 0.05), and O (γ_11_ = −0.035, *p* < 0.05) significantly moderate the relationship between psychological distress and communication disturbance: the lower the level of these country indicators, the higher the correlation between psychological distress and communication disturbance. The opposite direction of moderation can be observed in the relationship between psychological distress and phone obsession: the higher the GGI (γ_21_ = 0.042, *p* < 0.01), the SCI (γ_21_ = 0.038, *p* < 0.05), HN (γ_21_ = 0.038, *p* < 0.05), and O (γ_21_ = 0.046, *p* < 0.05), the higher the correlation between psychological distress and phone obsession.

## Discussion

The main aim of our exploratory study was to investigate the moderating role of country-level indicators in the relations between phubbing and psychological distress. It should be noted that the analyses of measurement invariance across countries for the psychological distress and phubbing variables showed only metric invariance (see Błachnio et al., 2021), which allowed us to compare countries in terms of correlations between variables. It did not, however, allow us to compare countries in terms of the levels of particular variables, which is the most common type of cross-cultural comparisons. This calls into question some of the studies to date that have compared similar variables across countries without testing measurement invariance.

To the best of our knowledge, previous studies have not considered the moderating role of cultural indicators in the relationship between phubbing and distress; therefore, we can only refer to cross-cultural comparisons, such as similar constructs (e.g., Balhara et al., 2019; Błachnio et al., 2019b; Panova et al., 2020). In our exploratory study, we tried to answer the question of whether selected country indices were related to phubbing. We also intended to establish if country indicators moderated the relationship between communication disturbance and psychological distress. To explore this issue, we chose indicators related to quality of life, namely, the WHI, the SPI, the HDI, and the GGI, which is an indicator of national gender gaps on several levels, and selected cultural dimensions distinguished by Hofstede.

In almost all countries we found a similar pattern: the higher the phone obsession and communication disturbance, the higher the psychological distress. In other words, both social and behavioural aspects of excessive mobile phone use are positively correlated with distress. In our study, the mean correlation between distress and communication disturbance was 0.21 (95% CI [0.17, 0.25]), while the mean correlation between psychological distress and phone obsession was 0.15 (95% CI [0.11, 0.20]). This finding is in line with previous studies, where psychological distress was related to phubbing (Liu et al., 2019) and significantly contributed to SNSs addiction (Pontes et al., 2018). More specifically, a meta-analysis by Marino et al. (2018) confirmed that problematic Facebook use and psychological distress were positively correlated.

The results of the present study indicate that the amplifying or weakening effect between phubbing and psychological distress is dependent on some cultural indicators. As predicted, the findings show that in almost every country communication disturbance results in increased distress, but this happens to a different extent in different countries. In countries with lower GGI, O, and HN, phubbing has more serious consequences for distress. For example, in Pakistan and India, the social context and the nonverbal aspects of communication, such as showing respect or disrespect toward the other party, are important (see Hall, 1990). Therefore, using a phone in the presence of another person will be perceived as ignoring the communication partner and result in the deterioration of social relationships. It is likely that in these countries, due to the prevailing system of values, the deterioration of social relationships with one person or a group of people cannot be compensated for by establishing a new and equally valuable relationship with others.

As expected, phone obsession correlated positively with distress in almost every country. However, in countries with higher GGI, SPI, O, and HN (e.g., Spain, Portugal, and Slovenia), phone addiction was associated with distress more strongly than in others. It is reasonable to assume that people in these countries are more dependent on phone availability to function in many life domains, which in turn may translate into higher levels of distress related to phone obsession. In contrast, this relationship was relatively weaker in countries with lower GGI, SPI, O, and HN (e.g., Pakistan, China, and Ukraine), which may be due to the fact that phone use in these countries tends to be more limited, and although phone obsession may be as strong there as in other countries, its effects on distress may be smaller.

It should be noted that these findings receive support from previous studies on the cultural correlates of Internet addiction (Błachnio et al., 2019b), where a high level of Internet addiction was related to the HDI. As accessibility to technologies varies across cultures (Jackson and Wang, 2013), higher Internet addiction has been reported in highly technologically developed countries. Our current study reveals a similar pattern. A great body of research has shown that problematic mobile phone use is associated with lower well-being and lower mental health indicators (e.g., Volkmer and Lermer, 2019).

## Limitations and Directions for Future Research

The present study is not free from limitations. Firstly, its cross-sectional design precludes any longitudinal inferences. Secondly, this was a correlational study, and it would therefore be unwarranted to draw causal conclusion. Thirdly, the study was based on self-report measures, which means we investigated subjective indicators of problematic mobile phone use. In future studies, it would be useful to incorporate the behavioural assessment of mobile phone use as well. Lastly, although we found robust results across countries, the study is limited to convenience sampling, which is why generalizing current research findings requires caution. It would be advisable to collect data from more representative samples for each country in the future.

Although we present results from 20 countries, it could be argued that they do not represent all cultures. Many researchers point to the problem of cross-cultural comparisons being conducted on WEIRD cultures (Western, Educated, Industrialized, Rich, and Democratic; Henrich et al., 2010). In future research, it is worthwhile to expand the number of countries and include more countries from outside the group called WEIRD cultures.

It is also worth noting the low Cronbach’s alpha for the Phone Obsession Scale in Serbia, though it should be mentioned that in shorter scales (up to 5 items) alpha larger than 0.65 is acceptable (Cortina, 1993).

## Conclusion

To sum up, the results of our study demonstrate the importance of measurement invariance testing, the results of which indicate what type of analysis and what type of conclusion are valid in a given cross-cultural comparison. We have found that psychological distress is related to communication disturbance and that, for the most part, this relation is culturally universal. Other studies also revealed a similar pattern of this relationship (e.g., Tekkam et al., 2020). However, the power of our findings stems from the number of culture-level indices included, which is a contribution this research makes to the current state of knowledge. The relationship between phubbing and psychological distress has some macro-level determinants (e.g., the GGI, which has been included in our research). The presented results shed light on the relationship between phubbing and psychological distress and its universality across cultures. A better understanding of phubbing may help in coping with its effects in the social fabric of communities, regardless of the cultural background. The current findings underline the importance of clinical awareness of problematic mobile phone use. Considering the omnipresent use of mobile phones as important tools in many aspects of everyday lives, it is important to identify the signs of psychological disturbances and to prepare adequate interventions. This study may be helpful for clinicians and therapists in designing programs targeting phubbing. Interventions addressing psychological distress are considered to be important across cultures when dealing with this problematic phenomenon.

## Data Availability Statement

The raw data supporting the conclusions of this article will be made available by the authors, without undue reservation.

## Ethics Statement

The studies involving human participants were reviewed and approved by The Institute of Psychology Ethics Committee (The Catholic University of Lublin). Written informed consent for participation was not required for this study in accordance with the national legislation and the institutional requirements. However, consent was implied *via* completion of the questionnaires.

## Author Contributions

AB: idea of the manuscript, preparing research, supervising research in all countries, and writing the manuscript. AP: preparing research and comments on final version of the manuscript. OG: statistical analyses, co-writing Materials and Methods section and writing Results section, preparing methods in Polish language, collecting data in Poland, and comments on final version of the manuscript. RB, MD, ESD, MB-E, and MB: preparing methods in their languages, collecting data in their countries, coding the data, and giving comments on whale stages of writing manuscript. MM: preparing methods in their languages, collecting data in their countries, coding the data, comments on final version of the manuscript, and English proofreading. AA, AMA, MJB, TB, NB, JG, JH, AI, SM, EM, AM, BM, IP, BR, GS, LD’S, MV, MW, AMSW, and SY: preparing methods in their languages, collecting data in their countries, coding the data, and comments on final version of the manuscript. All authors contributed to the article and approved the submitted version.

## Conflict of Interest

The authors declare that the research was conducted in the absence of any commercial or financial relationships that could be construed as a potential conflict of interest.

## Publisher’s Note

All claims expressed in this article are solely those of the authors and do not necessarily represent those of their affiliated organizations, or those of the publisher, the editors and the reviewers. Any product that may be evaluated in this article, or claim that may be made by its manufacturer, is not guaranteed or endorsed by the publisher.

## Footnotes

1 Power Distance reflects the way people view the degree of power distribution in their culture and the degree to which they accept their place in the social hierarchy. The individualism vs. collectivism dimension concerns the level of integration with the group, which shows whether the importance is placed on attaining personal or group goals. The Uncertainty Avoidance dimension reflects the degree to which societies tolerate unknown situations, changes, and unexpected events. Finally, the masculinity vs. femininity dimension reflects what kind of values are respected—for instance: assertiveness, ambition, and power are masculine values, whereas relationship building is central to feminine values.

2
https://worldhappiness.report

3
http://hdr.undp.org

4
www.hofstede-insights.com
